# Classification of colorectal tissue images from high throughput tissue microarrays by ensemble deep learning methods

**DOI:** 10.1038/s41598-021-81352-y

**Published:** 2021-01-27

**Authors:** Huu-Giao Nguyen, Annika Blank, Heather E. Dawson, Alessandro Lugli, Inti Zlobec

**Affiliations:** 1 Institute of Pathology, University of Bern, Murtenstrasse 31, 3008 Bern, Switzerland; 2 Institute of Pathology, Triemli City Hospital, Birmensdorferstrasse 497, 8063 Zurich, Switzerland

**Keywords:** Image processing, Machine learning, Classification and taxonomy, Colorectal cancer

## Abstract

Tissue microarray (TMA) core images are a treasure trove for artificial intelligence applications. However, a common problem of TMAs is multiple sectioning, which can change the content of the intended tissue core and requires re-labelling. Here, we investigate different ensemble methods for colorectal tissue classification using high-throughput TMAs. Hematoxylin and Eosin (H&E) core images of 0.6 mm or 1.0 mm diameter from three international cohorts were extracted from 54 digital slides (n = 15,150 cores). After TMA core extraction and color enhancement, five different flows of independent and ensemble deep learning were applied. Training and testing data with 2144 and 13,006 cores included three classes: tumor, normal or “other” tissue. Ground-truth data were collected from 30 ngTMA slides (n = 8689 cores). A test augmentation is applied to reduce the uncertain prediction. Predictive accuracy of the best method, namely Soft Voting Ensemble of one VGG and one CapsNet models was 0.982, 0.947 and 0.939 for normal, “other” and tumor, which outperformed to independent or ensemble learning with one base-estimator. Our high-accuracy algorithm for colorectal tissue classification in high-throughput TMAs is amenable to images from different institutions, core sizes and stain intensity. It helps to reduce error in TMA core evaluations with previously given labels.

## Introduction

Over the last decades, Tissue Microarrays (TMAs) have become synonymous with translational research. Not only used as a high-throughput screening tool for different tissue biomarkers (protein, RNA or DNA)^1^, they can also be generated in ways to study tissue heterogeneity^2,3^, as well as to establish new antibodies or set up new experimental methods (e.g. CODEX)^4,5^. TMAs are important components of clinical trials and biobanks, and together with detailed clinicopathological patient data can be a sustainable research tool.

With digital pathology at our doorstep, TMA archives can now also be used as a way of generating training and testing data for computational algorithms. Well-labeled TMAs with both image and patient annotations can potentially provide a hugely valuable resource for downstream algorithm development^6^. Additionally, TMAs generated on the basis of digital scans can allow users to label histopathological areas of interest directly on the images using various annotation tools (sizes and diameters)^7^. The annotated scans can then be aligned to the corresponding tissue blocks, which are cored out and transferred to a recipient TMA block. From the computational point-of-view, the advantage of TMA construction using this approach are many. Not only are targeted tissue areas cored out, allowing researchers to investigate even small or complex histopathological areas across hundreds of patient samples effectively (e.g. tumor budding)^8^ but whole slide images are immediately annotated by the experts, thus creating high-quality labels, which can be exploited in further analysis. Moreover, TMA cores will be assigned labels of the expected tissue core content, based on the whole slide image annotation. The use of digital scans to target specific research questions is an integral component of the so-called “next-generation Tissue Microarray (ngTMA)” approach, which has been used by our research group since 2012^7^. Since then, a massive archive of tumor/normal tissue ngTMAs has been generated along with their corresponding single-core images after H&E or immunohistochemistry staining.

Despite these advantages, TMAs are not without some drawbacks^9^: serial sectioning of the TMA block will inevitably lead to shifts in the expected tissue content within each core. For example, a TMA constructed to contain only cancer of a certain type may, after numerous sections contain fewer cores with tumor epithelium and many more with only stroma, or normal tissue. This is a usual “side effect” of TMAs due to the three-dimensionality of the tissue core and may be more or less severe depending on tissue type, tissue thickness and number of times the TMA block was sectioned. The shift in tissue content through this “z-axis” requires re-labelling of each tissue core in order to avoid any error carried through into next steps, a task which can be tedious, especially with high-throughput TMA sets containing thousands of tissue cores.

Colorectal cancers (CRC) are excellent candidates for construction of such high-throughput TMA. CRCs are heterogeneous on the histopathological and molecular level^10,11^. They exhibit a complex microenvironment of tumor-stroma interactions/components linked to the aggressiveness of the cancer^12^. The sum of these parts is to a large degree represented by the histopathological image. From a clinical perspective, CRC remains a significant burden with low 5-year survival rates^13^. A “personalized medicine” approach to CRC treatment is still largely missing. With computational methods, the image itself has the potential to become a new “biomarker” and TMA archives containing well-labeled CRC tissues and corresponding patient data may provide the necessary input to unravel the complexities of this disease.

The aim of this study is to develop a colorectal tissue classifier discriminating between colorectal tumor tissue, normal tissue and other tissue types, applied to high-throughput tissue microarrays. This TMA pipeline allows one to re-label colorectal tissue cores for future biomarker analysis. To this end, we use three different international cohorts, totaling 54 ngTMA slides and more than 15′000 tissue cores stained with H&E as well as a computational approach with different ensemble CNN methods.

## Methods

### Patient cohorts

Seven hundred and seventy patients from three different cohorts were entered into this study including 410 patients from Switzerland, 89 patients from Germany and 271 patients from Canada. All patients were diagnosed with primary CRC: cohorts from Switzerland and Germany were stage I-IV, while that from Canada included only patients with stage II disease. Patient characteristics have previously been described and are found in Supplemental Table 1.

### Tissue microarray collection

ngTMAs from all cohorts have previously been constructed^14^. Using archival formalin-fixed paraffin-embedded tissue blocks from corresponding pathology archives, freshly cut H&E or pan-cytokeratin stained slides were digitally scanned. Using a tissue microarray annotation tool, areas of tumor center, invasion front, tumor stroma, and normal colonic tissue were marked on the digital image using a 0.6 mm (Switzerland and Canada) and 1.0 mm (Germany) diameter annotation tool. Multiple areas were marked for each primary CRC. Using a TMA Grandmaster instrument (3DHistech), tissue blocks and digital scan were aligned and the correspondin g annotated areas punched out and transferred into a recipient ngTMA block. The details of optical parameters used for scanning the slides of all three cohorts is shown in Supplemental Table 2. For the Swiss cohort, triplicates of all ngTMA blocks were made. For all ngTMAs, an H&E section was generated and scanned. This resulted in 54 different ngTMA slides totaling 15,150 tissue cores for analysis in this project. The use of patient data and tissue have previously been approved by the Ethics Committee of the Canton of Bern, Switzerland (KEK2017-01783) and other corresponding ethics committees. All relevant guidelines of the Institute of Pathology, University of Bern, Canton of Bern, Switzerland were followed for the study. The informed consent was obtained from all subjects or, if subjects are under 18, from a parent and/or legal guardian. At the time of ngTMA construction, similar tissue types, i.e. normal epithelium, tumor epithelium or tumor stroma, were pooled onto the same TMA recipient block, therefore prior knowledge with regard to the expected content of the tissue punch was available.

### ngTMA H&E tissue core extraction

In order to produce a single image from the larger ngTMA scan, we find the contour of the TMA core at lowest level of image resolution by applying a gray intensity threshold on the Gaussian blurring and smoothing image. The bounding box of these contours locates the core in the image. The “control” cores, which are typically placed inside the TMA block are removed based on their locations and the number of cores in the TMA row. In our dataset, each TMA image contains an average 289 cores detected by this solution. Supplemental Fig. 1 shows an example of the TMA tissue core extraction process.

### Color enhancement

Because of the diversity of the image dataset (different institutes of pathology, different pre-analytical variables and slide scanners/versions), the quality of images was variable in terms of brightness/darkness. For example, the images of the Canadian cohort are of high average brightness and give a much lighter image compared to Swiss and German cohorts. Practically, traditional approaches try to normalize the color space of data by estimating a color deconvolution matrix to identify the underlying stains^15,16^ or generating the new color using deep learning itself^17^. In this study, we want to keep the realistic variations of data and apply color augmentation to avoid the neural network generalization error in brightness, contrast perturbation. We consider the adaptive gamma correction for image contrast enhancement based on cumulative distribution function (CDF) of the pixel gray levels with in image itself^18^. An example of color enhancement can be seen in Supplemental Fig. 2.

### Ensemble methods for TMA core classification with two effective architectures: VGG16 and CapsNet

#### Image extraction and augmentation

The input of these neural networks is the TMA core at level *l* of image resolution. We then extract the overlapping tiles with size of 224 × 224 and an overlap factor *f*. To provide the experiment results in this study, our choices are l = 3 and f = 0.3 by optimizing hyperparameters with random search. Then, we apply data augmentation including rotation, shift, shear, zoom, flip as well as color to increase variability and reduce over-fitting of data. Each neural network independently classifies the *224* × *224* H&E staining image tiles into the three tissue classes: tumor, normal epithelium or other tissues (including: fat, stroma, muscle, blood, artifacts, etc.).

#### CNN architectures

In practice, CNN architectures have been explored by reformulating different activation and loss functions, parameter optimization, regularization, and structural innovations^19^. In this study, we tried to hand-tune for finding the right configuration with our specific data and requirements. We considered the classical VGG16 architecture^20^ which has achieved excellent performance in image classification by directly learning useful features from the training image patches and optimising the loss function^21–23^. It consisted of 13 convolutional layers, 5 max pooling, dropout and 3 fully-connected layers. The main drawback of the CNN-based architecture is that it does not take into account the location and spatial relations between the entities in images because of max-pooling operations. But the relation of small objects in histopathology images plays an important role. Therefore, we consider the capsule network with dynamic routine proposed by Hinton et al.^24^. Theoretically, a capsule is a nested set of neural layers which outputs a vector (not a scalar likes VGG16) to represent various properties of objects such as position, orientation, skewness, scaling, orientation, translation and so on inside an image to define the probability of some entity's existence. The most significant characteristic of CapsNet is routing by agreement (instead of using max-pooling layer of VGG16), i.e. the capsule makes the prediction of the capsules in higher hierarchical levels and then these parent capsules will be activated. It consisted of 4 layers: a feature extraction with conventional layer followed by ReLU activation, a primary capsule layer with ’squash’ activation, a capsule layer with routing by agreement algorithm, a reconstruction layer used to predict the output. Here, the hyperparameter of both neural network architectures: VGG16 and CapsNet were selected by random search.

### Five classification flows

**Figure 1:**
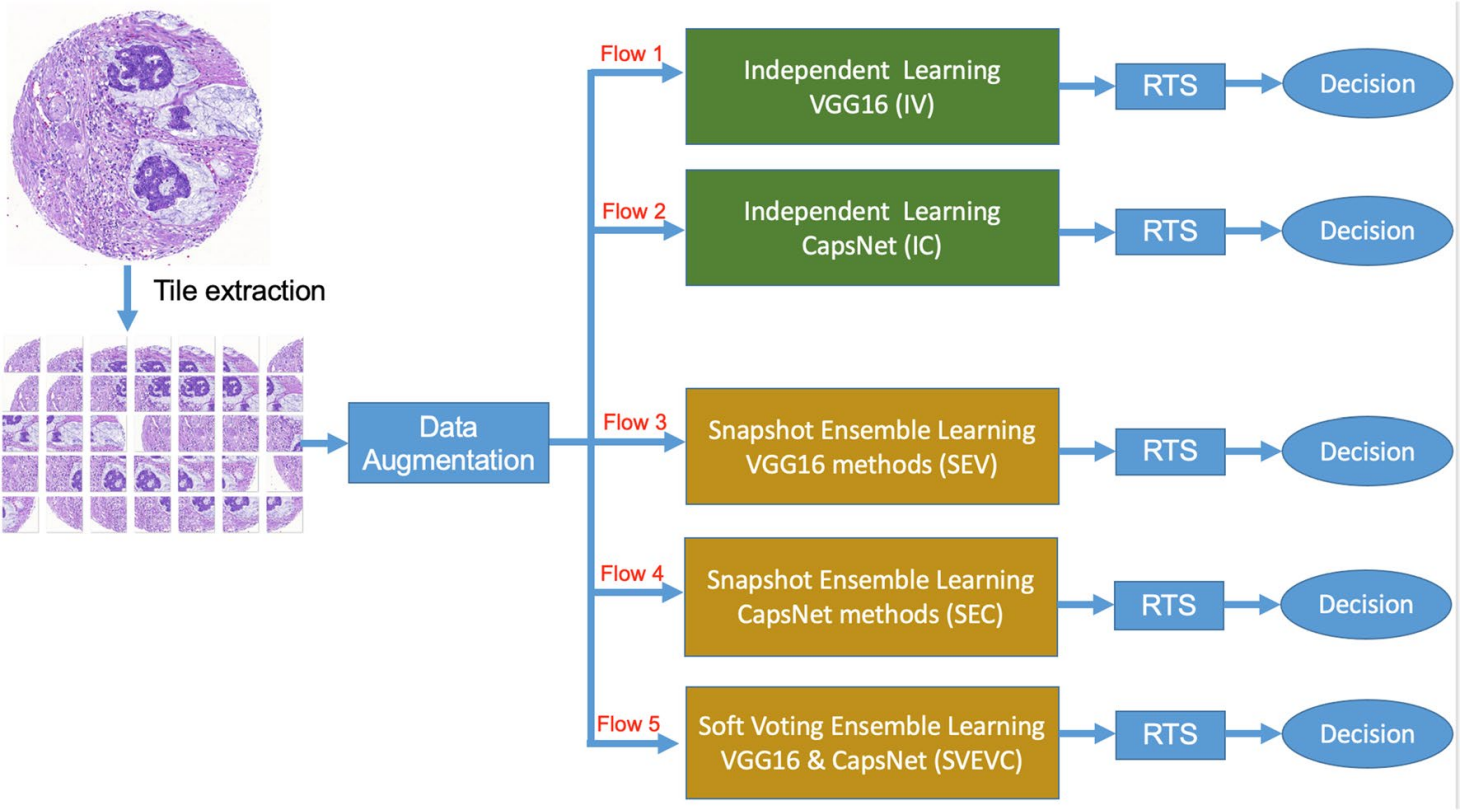
The scheme for TMA core classification considered in this study with five different flows for independent and ensemble learning with VGG16 and CapsNet methods. The inputs of these flows are the tiles extracted from each TMA core with data augmentation. We apply Random Transformation Sampling (RTS) to refine the uncertain cases of deep learning methods.

Figure 1 shows the scheme of TMA tissue core classification using the deep learning techniques. We consider 5 different flows for the classification prediction with two single models and three types of ensemble models. An augmentation for test image using RTS is applied for all of these flows. In the two-first classification flows, each neural network (VGG16 and CapsNet) independently trains and makes the prediction for all tiles extracted from the TMA image. Three other classification flows are based on ensemble methods to train multiple models and combine their predictions. The key benefit of ensemble learning is in improving the performance of predictions by limiting the sensitivity of specific training data, of training scheme, and the serendipity of a single model. Flow 3 (and Flow 4) work with a base-estimator VGG16 (respectively, CapsNet) using Snapshot Ensemble^25^. Flow 5 applies Soft Voting Ensemble^26^ to reduce the error rate by training different base-estimators (VGG and CapsNet).

### Ensemble methods

Many techniques for ensemble methods have been proposed in the literature, for example Bagging^27^, Adaboosting^28^, negative correlation learning^29,30^ and their variants. These methods have become very prominent by ensuring the reliability and stability of the predictions of models. However, a limitation of ensemble deep learning methods is that it often requires the large computational cost of training with big data and multiple models. Therefore, we consider the Snapshot ensemble method developed by Huang et al.^25^. Essentially, the idea of Snapshot Ensemble is to train multiple model snapshots during a single training run and combine their predictions to make an ensemble prediction. In practice, we use a training budget of 200 epochs; i.e. 5 model snapshots of 40 epochs. Instead of training many neural networks from scratch, a Snapshot Ensembling has an optimization process which visits several local minima before converging to a final solution, saving the weights and adding the corresponding network to ensemble. A Snapshot Ensembling uses the cosine annealing schedule as an aggressive learning rate schedule. The learning rate of the first training epoch of a cycle starts with the maximum value (set to 0.1) and is dropped relatively rapidly to a minimum value near zero before reset again in the next cycle to escape the current local minima and attenpt to find another possibly better one. At test time, the prediction is based on the average of the last 2 model’s softmax outputs. On the other hand, we apply Soft Voting Ensemble^26^ for multi base-estimators (here, one VGG and one CapsNet) in order to balance out their individual weaknesses using the average probabilities over predictions from all base estimators.

### Uncertain cases and decision making in histopathology images

VGG16 and Capsnet can provide a powerful mapping for the visual features of input image to an output prediction with both types of learning: single and ensemble. However, because the test data contains a diversity of unseen tumor types and artifacts with the staining and scanning differences, the measure of uncertainty is a need for classification decision making^31^. We apply RTS for uncertainty quantification to the test images in an inference step. A set of random transformations with brightness, color, contrast, sharpness, rotation, shift, shear, zoom in/out, and translate X/Y was added on test images. Each transformed image is processed to obtain a prediction. Considering all outputs $f$ of $t$ random transformations $\phi$, the mean $\mu$ and the variance $\sigma$ provides the refined prediction and the uncertainty score of a test image $x$ which is classifed into a class $j$ as follow:

$$\mu_{j} = \frac{1}{t}\mathop \sum \limits_{i = 1}^{t} \left( {f_{j} \left( {\phi_{i} \left( x \right)} \right)} \right)\quad \sigma_{j} = \frac{1}{t}\mathop \sum \limits_{i = 1}^{t} \left( {f_{j} \left( {\phi_{i} \left( x \right)} \right) - \mu_{j} } \right)^{2}$$

For each TMA core, we extract the overlapping $n$ tiles. For all of five deep learning classification flows, the output information of a TMA core for each class $j$ includes:

- the number of best classification prediction: ${BP}_{j}=coun{t}_{i=1}^{n}(j==argmax({\mu }_{1},{\mu }_{2},{\mu }_{3}))$
- the average of prediction score: ${PS}_{j}=\frac{1}{n}\sum_{i=1}^{n}({\mu }_{j})$
- the average of uncertainty score: ${US}_{j}=\frac{1}{n}\sum_{i=1}^{n}({\sigma }_{j})$

The classification decision of each TMA core is based on the class with the largest ${BP}_{j}$ score over all its tiles. If there are two classes with similar scores, we will compare the second criteria: ${PS}_{j}$ score (bigger is better) and then the third criteria: ${US}_{j}$ score (smaller is better). In practice, we set a threshold for the differences of ${PS}_{j}$ score (${\varepsilon }_{1}=1\%)$ and ${US}_{j}$ score (${\varepsilon }_{2}=0.1)$ among the classes. If these three criteria can not provide a clasification decision for a TMA score using deep learning, we call the uncertain classification result. This decision-making rule is directly applied for the ngTMA slides with mixed contents of the punch. Here, the German cohort of our data has not the general label. As mentioned, ngTMA slides were often constructed with an assigned general label of the expected tissue core content based on the whole slide image annotation, such as tumor center, tumor front, stroma and normal tissue. All of TMA slides of Swiss and Canadian cohorts have this prior innformation. The final classification decision for these cohorts is the class where the deep learning flow provides the prediction as similar information with prior content of the punch. Otherwise, we also call the uncertain classification result. Supplemental Fig. 3 shows some examples of uncertain cases. All of these uncertain cases will be highlighted and should be re-checked by pathologist experts in the validation step. The steps of this study are outlined in Supplemental Fig. 4.

## Study design

### Ground-truth annotation

The class annotation of each TMA core image was checked by two pathologists (AB, AL). Almost all TMA cores were easily classified into three classes: tumor, normal, and other tissues. However, a subgroup of tissue cores caused difficulty in the classification by our pathologists and these cores were eliminated from any analysis (n = 496; 3.2%). In total, 15,150 TMA cores with annotation were used for training, validation and testing.

### Training, validation and testing data

We balanced this data into training set and testing set, using three ngTMAs labeled for normal epithelium, two expected to contain stromal areas and three containing tumor epithelium from the center or invasion front, resulting in eight ngTMAs for training and validation (n = 2144 cores, 14.15% of all available cores). For training of the algorithm, we split 25% of the data for validation, 75% for training. The remaining images were used for testing (46 slides with 13,006 cores, 85.85% of all available cores). We use two different scenarios for the evaluation of the classification performance:

- Evaluation A: We evaluate the best algorithm (SVEVC) using the manual verification of pathologists. We asked both pathologists to randomly select 16 slides (totaling 4317 cores) from 46 testing slides for this evaluation.
- Evaluation B: We used the remaining 30 slides of testing dataset for the comparison of the algorithms to the ground truth.

The more detail of number of slides and cores of each cohort using for training and two evaluation scenarios is in Supplemental Table 3.

### Evaluation metrics

For quantitative evaluation of the classification, we counted the output prediction of each class for each method compare with ground-truth: True Positive (TP), True Negative (TN), False Positive (FP), and False Negative (FN). Then, we computed some basic statistical measures as follow:

- $$Recall=\frac{TP}{TP+FN}$$
- $$Precision=\frac{TP}{TP+FP}$$
- $$F1Score=\frac{2*TP}{2*TP+FP+FN}$$
- $$Accuracy=\frac{TP+TN}{TP+TN+FP+FN}$$

For quantitative evaluation of the paired differences between the output of deep learning flows on same test images, we apply the non-parametric Wilcoxon signed-rank test. In addition, an adjustment for multiple hypothesis testing using the Bonferroni method was used. Considering eight tests, only *p* values < 0.00625 are found to be statistically significant. All tests were two-sided.

## Results

**Figure 2:**
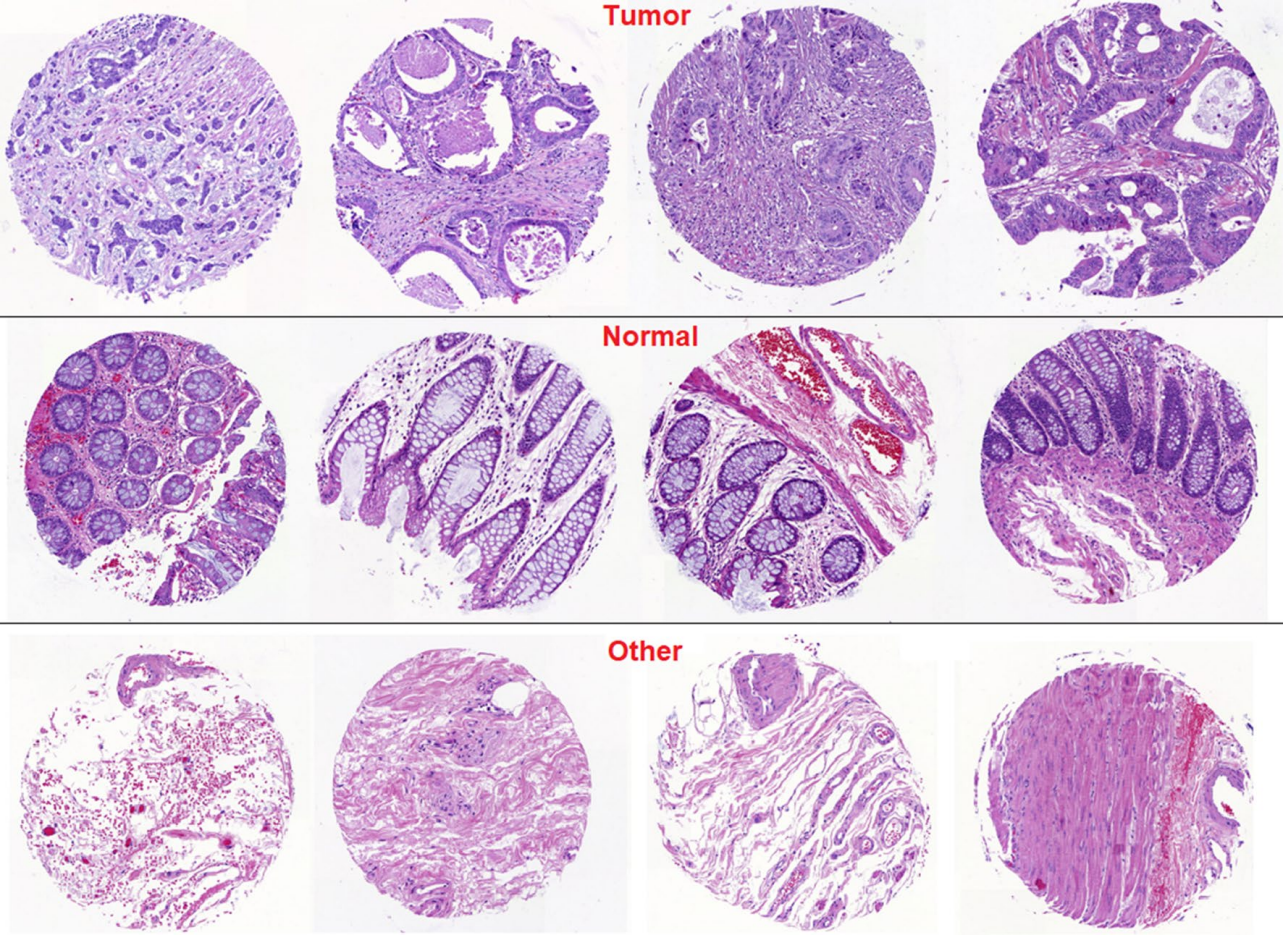
Examples of correct classification for each tissue classes.

Considering the evaluation B: comparison of the algorithms to the ground truth, we applied all five deep learning flows on a further 8689 TMA cores from 30 slides, where 21 slides, 4 slides and 5 slides were punched in the area of tumor center, of normal colonic tissue and of tumor stroma, respectively. We compared the classification prediction output with the grouth-truth. Figure 2 shows some examples of correct prediction outputs.

### Comparison of different TMA tissue classes

**Figure 3:**
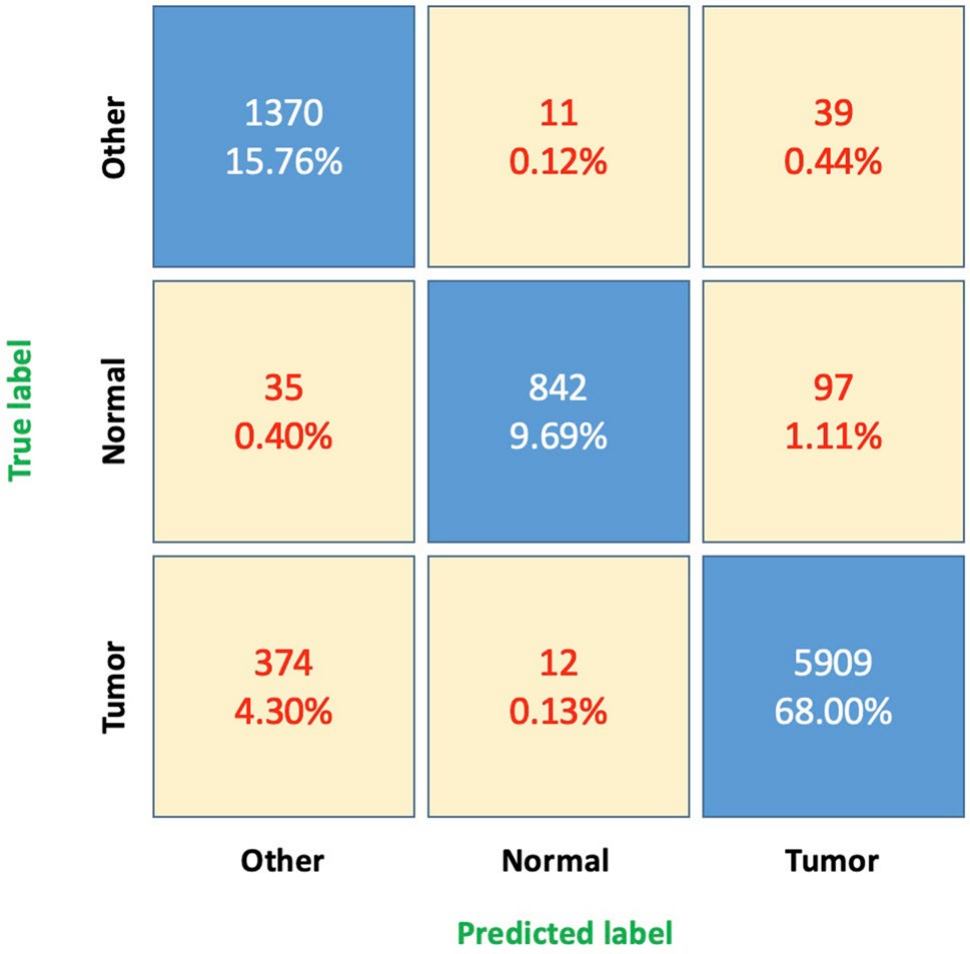
Confusion matrix showing misclassification errors across tissue types using SVEVC method in evaluation B.

**Figure 4:**
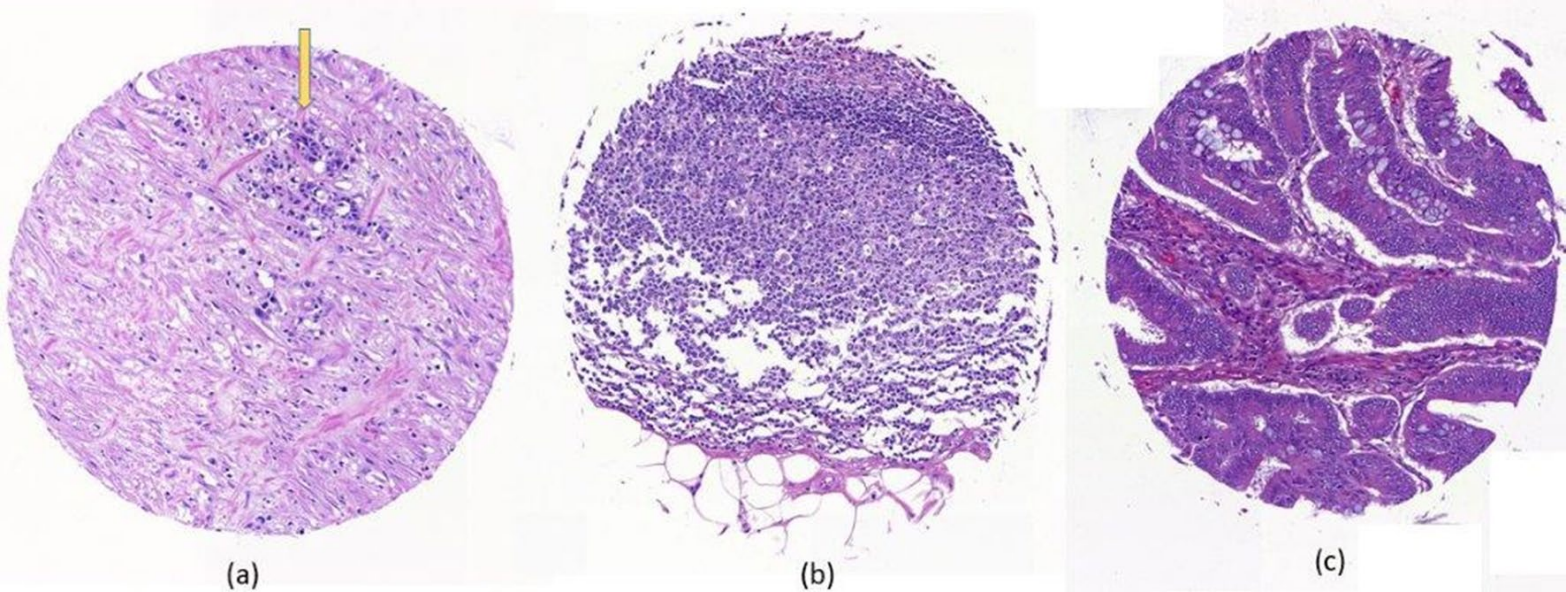
Example of some error classification where: (**a**) the case of small tumor area (yellow arrow) was predicted as other, (**b**) unseen tissue of complex tumor stroma was predicted as other, (**c**) adenoma case was predicted as tumor.

Table 1 shows the result detail of evaluation B for True Positive, False Positive, True Negative, False Negative and Recall, Precision, F1-score, Accuracy of each tissue classes for all of five flows proposed: IV (Independent learning with VGG16), IC (Independent learning with CapsNet), SEV (Snapshot Ensemble learning with VGG-based), SEC (Snapshot Ensemble learning with CapsNet), SVEVC (Soft Voting Ensemble learning with one VGG-based and one CapsNet). Considering accuracy values, the class of normal tissues with SVEVC obtains the highest score with 0.982 compared to 0.939 and 0.947 of tumor and other tissues, where the overall of accuracy of SVEVC is 0.956. Figure 3 highlights the confusion matrix of classification, which helps to understand which tissue is being mistaken for another tissue-type. We observe the highest error rates are in two cases: Case 1: 374 tumor cores were predicted as other and Case 2: 97 normal cores were predicted as tumor (Fig. 4). Almost all prediction errors in Case 1 are explained by the low composition of tumor tissue compared to the area of other tissues or by some complex tumor stroma tissues unseen in training. In Case 2, we observe polyp cases wrongly classified into the tumor class.

**Table 1:** The detail of statistical measures of five proposed deep learing classifications reported with 30 slides in evaluation B.

	TP	FP	TN	FN	Recall	Precision	F1-score	Accuracy
**Tumor**								
*IV*	5615	186	2199	548	0.911	0.967	0.938	0.914
*IC*	5766	198	2184	491	0.921	0.966	0.943	0.920
*SEV*	5755	162	2234	480	0.923	0.972	0.947	0.925
*SEC*	5879	174	2210	438	0.930	0.971	0.950	0.929
*SVEVC*	5909	142	2252	386	0.938	0.976	**0.957**	0.939
**Normal**								
*IV*	820	137	7432	159	0.837	0.856	0.847	0.965
*IC*	806	83	7585	165	0.830	0.906	0.866	0.971
*SEV*	831	149	7503	148	0.848	0.847	0.848	0.965
*SEC*	807	105	7632	157	0.837	0.884	0.860	0.969
*SVEVC*	842	23	7692	132	0.864	**0.973**	0.915	**0.982**
**Other**								
*IV*	1283	507	6635	123	0.912	0.716	0.802	0.926
*IC*	1301	485	6743	110	0.922	0.728	0.813	0.931
*SEV*	1313	422	6792	104	0.926	0.756	0.833	0.939
*SEC*	1329	408	6873	91	0.935	0.765	0.841	0.942
*SVEVC*	1370	403	6866	50	**0.964**	0.772	0.858	0.947
**Overall**					**Average**			
*IV*	7718	830	16,266	830	0.887	0.847	0.862	0.935
*IC*	7873	766	16,512	766	0.891	0.867	0.874	0.940
*SEV*	7898	733	16,529	733	0.899	0.859	0.876	0.943
*SEC*	8014	687	16,715	687	0.901	0.873	0.884	0.947
*SVEVC*	8121	568	16,810	568	***0.922***	***0.907***	***0.910***	***0.956***

We highlight (in bold) the best results of: Recall, Precision, F1-score and Accuracy grouped by tissue classes and average of all classes. It shows that the SVEVC method obtains the best classification performance.

### Comparison of independent and ensemble learning

**Figure 5:**
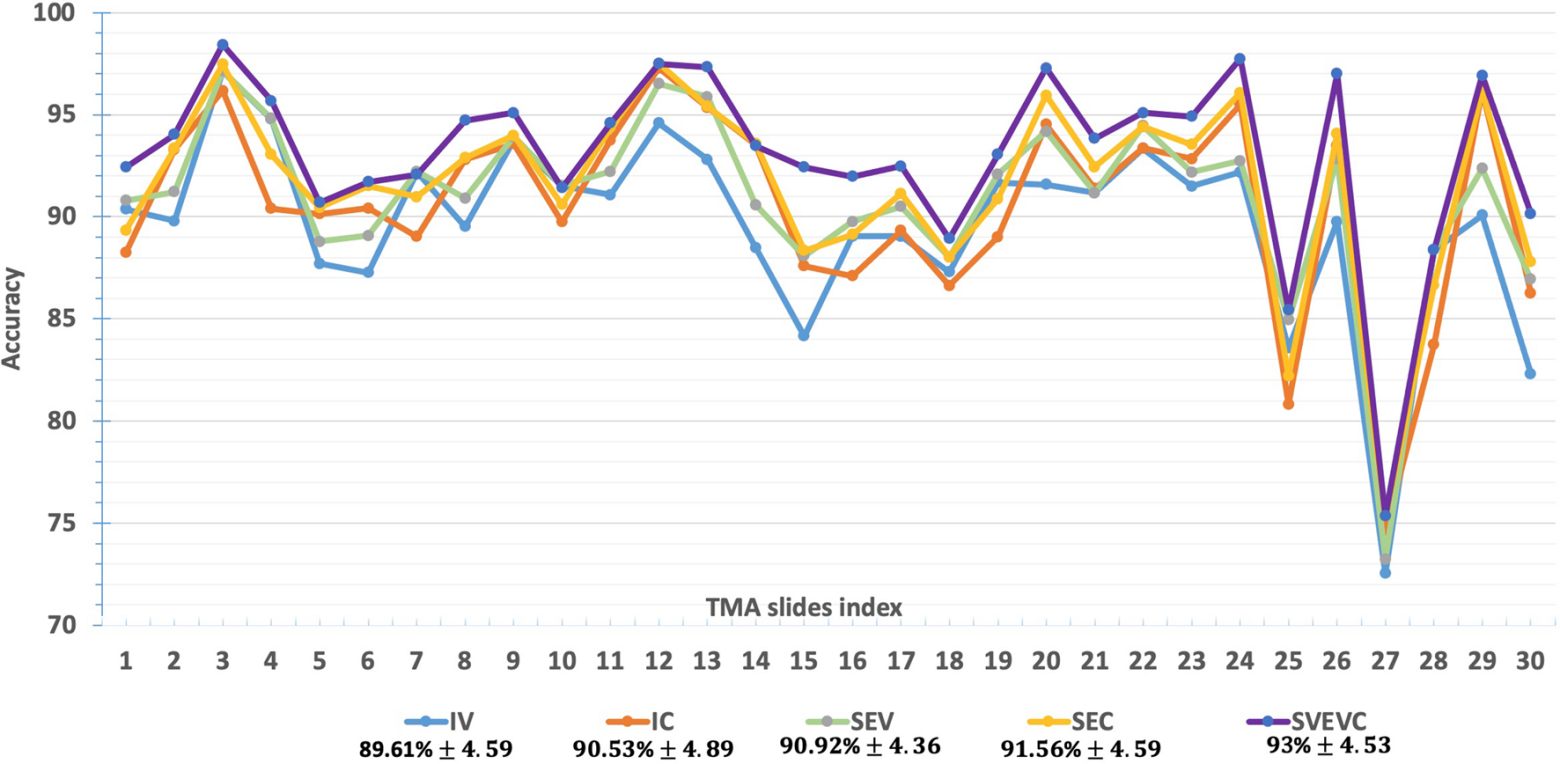
The detail result of the accuracy classification of five proposed flows on each TMA slide in evaluation B. The measures of mean and standard deviation per each method for 30 slides are given.

Figure 5 shows the detail of the accuracy classification of all five learning flows: IV, IC, SEV, SEV, and SVEVC on each TMA slide for all type of tissues. Overall, our proposed SVEVC approach reaches up to $93\%\pm 4.$ 3 of accuracy classification with a great improvement of around 2.5% (and 3.5%) compared with single learning IC (and IV) with $90.53\%\pm 4.89$ (and $89.61\%\pm 4.59$ respectively), also of around 1.5% (and 2%) compared, with ensemble learning using one base-estimator SEC (and SEV) with $91.56\%\pm 4.59$ (and $90.92\%\pm 4.36$ respectively). Supplemental Fig. 5 shows the detail of the number of correct classifications of each tissue type using the five learning flows. Table 2 shows the statistical p-value of nonparametric Wilcoxon signed-rank test. Considering these two single learning flows (IV versus IC), there is no significant improvement between them for all of the classes. When we applied ensemble learning, our findings highlight the improved predictions for tumor and other classes compared to independent learning method, with ${p}_{IV vs. SEV}= 4.15\mathrm{e}-05$ and ${p}_{IC vs. SEC}=8.35\mathrm{e}-06$ for Tumor class; ${p}_{IV vs. SEV}=0.0036$ and ${p}_{IC vs. SEC}=0.0020$ for “other” class. Taken together across five learning flows, we obtain an outperformance classification using the SVEVC versus three other methods: IV, IC, SEV for tumor class and versus all of four others (IV, IC, SEV, SEC) for “other” class. However, there is no difference observed for the very structured class, i.e.: normal tissue, when we consider the statistical p-value between different learning methods in this study.

**Table 2:** The statistical p-value after testing the null hypothesis between different learning flows: IV, IC, SEV, SEC, and SVEVC considering three classes of TMA tissues and uncertain predictions in evaluation B.

	Tumor	Normal	Other	Uncertain cases
IV versus IC	0.0156	0.2932	0.625	0.0235
IV versus SEV	**4.15e−05**	0.3711	**0.0036**	**8.75e−05**
IC versus SEC	**8.35e−06**	1	**0.0020**	**3.82e−05**
SEV versus SEC	**0.0031**	0.1422	0.4636	0.0227
IV versus SVEVC	**4.52e−05**	0.2807	**0.0002**	**0.0048**
IC versus SVEVC	**1.89e−05**	0.0421	**0.0001**	0.0133
SEV versus SVEVC	**0.0007**	0.5896	**0.0033**	0.0707
SEC versus SVEVC	0.1103	0.0417	**0.0030**	0.3448

Using *p* values < 0.00625 as the statistically significant level of difference, we highlight the improvement of prediction cases in bold.

### Uncertain cases evaluation

**Figure 6:**
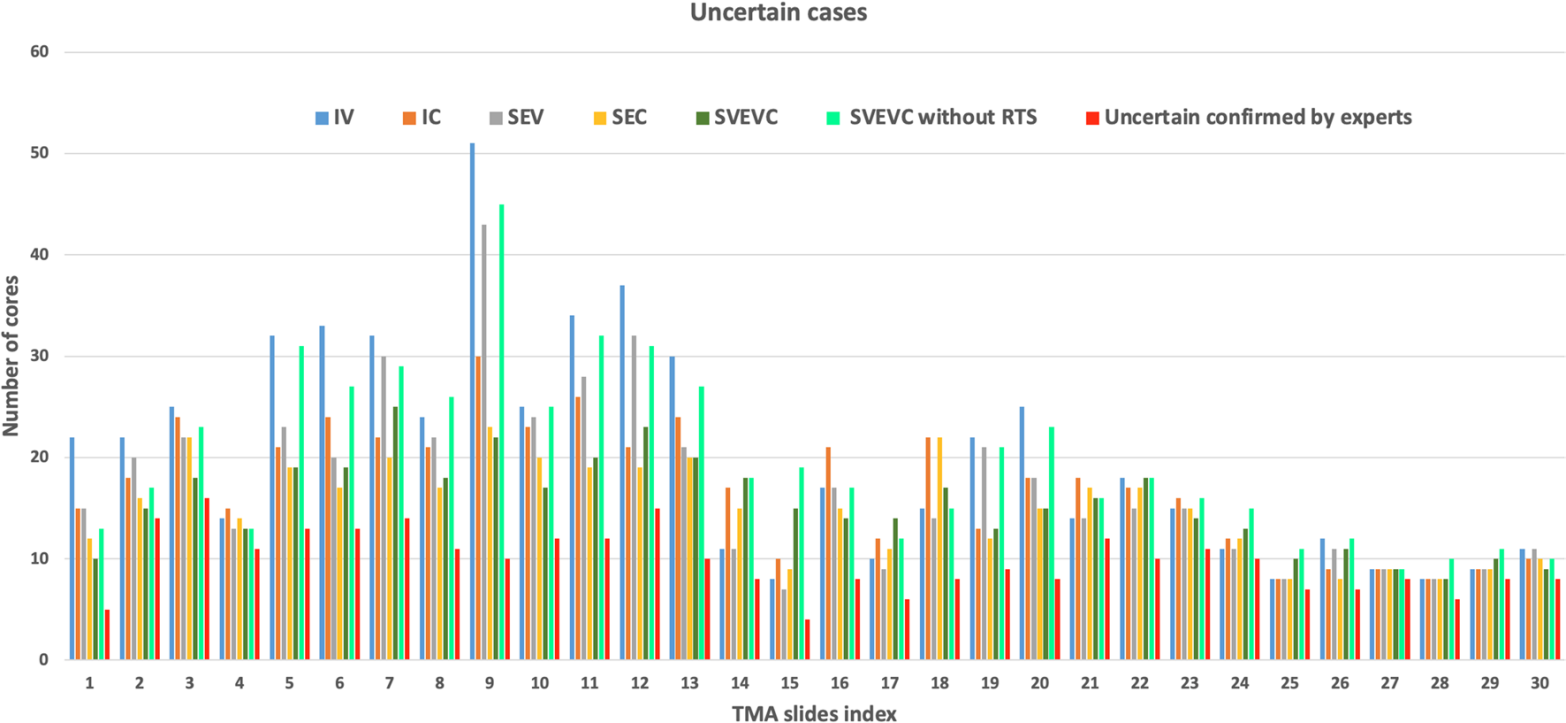
The number of uncertain cores of each slide confirmed by pathologists and predicted using five ensemble learning flows and the SVEVC methods without applying RTS method in evaluation B.

Figure 6 shows the detail of the number of uncertain cores reviewed by pathologist experts and predicted with five deep learning flows, including the SVEVC method without applying the RTS method in evaluation B. Here, without RTS method, a test image of SVEVC method will provide only one prediction score. Therefore, the final decision of a TMA core is based on two criteria: the number of best classification prediction (${BP}_{j}$) and then the average prediction score (${PS}_{j}$ with same threshold of difference ${\varepsilon }_{1}=1\%$) over all tiles of this TMA core. If there are two classes with similar scores for these two criteria, we call the uncertain classification result. The SVEVC method with RTS significantly reduces the number of uncertain predictions with $p=5.23E-05$ compared to the SVEVC method without RTS. The SVEVC method (with RTS) is the best method to limit the uncertain case in the classification decision compared to all other deep learning flows. In Table 2, we obtain the improvement for the detection of uncertain cases between the independent learning methods and ensemble learning methods with ${p}_{IV vs. SEV}=8.75\mathrm{e}-05$, ${p}_{IC vs. SVEVC}=3.82\mathrm{e}-05$ and ${p}_{IC vs. SVEVC}=0.0048$.

### Verification measurement by pathologists

Considering the evaluation A, we asked both pathologists to verify the classification prediction of the best performance learning flow SVEVC for 4317 TMA cores from 16 slides of Swiss cohort and German cohort (see Supplemental Table 3). Knowing that each TMA slide should contain a tissue type of interest, they see in one glance the incorrectly predicted individual TMA cores. These cores correspond either to cases that are wrongly classified (error cases) or cases with not enough information for a decision (uncertain cases) from their point-of-view. Other cases are considered correctly classified (Fig. 2). Supplemental Fig. 6 shows the pathologists’ decisions in detail. Considering the classification grouped by the different classes, the mean and standard deviation of classification accuracy using SVEVC method per slide is 91.96% + 2.07; $94.33\%\pm 3.13$ and $94.88\%\pm 1.27$ for normal tissues, tumor tissues, and other tissues respectively. Overall, the accuracy of classification using our SVEVC algorithm is $92.71\%\pm 3.51$.

### Comparison of different cohorts

Grouping the result by different cohorts for the whole testing datatset (46 slides, 13,006 TMA cores), we report here the mean and standard deviation of correct classification of SVEVC method per slide. The Swiss cohort reaches up to $93.46\%\pm 2.92$, compared to $90.13\%\pm 1.25$ for the Canadian cohort, while the German cohort has the classification performance of $90.97\%$ with 1 slide. The number of error prediction cores is 848, 102, and 14 for Swiss, Canadian, and German cohort, respectively.

## Discussion

In this study, we have generated a pipeline for the classification of colorectal tissues (tumor, normal or stroma/others) on high-throughput TMAs using ensemble deep learning methods with two neural network architectures (VGG16, CapsNet). We achieve an accuracy of 93–98% for all classes and demonstrate the outperformance of ensemble learning with two base-estimators SVEVC compared to an independent learning method.

A major aim of this study was the classification of tumor versus normal tissues and other tissue types, such as muscle, or stroma on high-throughput colorectal TMAs. Similar work has been performed by others for detection of different tissue types within CRC images. For example, using whole slide images (WSI), Kather and colleagues have generated a publicly available dataset of images including nine different tissue classes with corresponding labels from a set of 500 stage I–IV patients^22^. Shapcott and colleagues extracted images of CRC from the TCGA dataset and determined that sampling of smaller patch images from the larger whole slide results in similar features or clinicopathological associations whilst reducing computation time^32^. Yue et al. used unsupervised discriminative patch selection and the convergence of cluster level trained CNNs to infer prognosis of patients with stage I–II CRC^33^. Working on molecular subtyping, the group of Koelzer using deep learning methods for the identification of Consensus Molecular Subtypes (CMS) on H&E WSI. Their work establishes the H&E image as a surrogate for the CMS group^34^.

We focus here, however on TMAs. Such studies, especially in CRC using deep learning are still few. In CRC, Bychkov and colleagues performed a CNN-based analysis of 420 patients in order to stratify prognostic risk and show their result goes beyond what could be achieved using standard histopathology assessment^21^. In prostate cancer, TMA cores were used to train algorithms for Gleason pattern detection^35^, while in breast cancer, TMA samples were obtained to train an unsupervised classifier for survival (low versus high digital risk scores) compared to human evaluation of other features^36^. Finally, Fuchs and colleagues used TMAs to train random forest classifiers to detect cell nuclei in renal cell cancer and demonstrate the performance of a computational approach as compared to human evaluation^37^. Here, we go one step further: we used a large patient cohort and image number (> 15,000) to train, test and validate an algorithm for detection of colorectal tissue from extracted single TMA core images originating from different institutes at different times and prepared with different punch sizes.

It is common practice to perform a “second look principle” to confirm a diagnosis of cancer, as in our own pathology institute. Therefore, when building an AI system for histopathology analysis, using ensemble learning of different deep learning methods mimics this process and helps to avoid the risk for decision making of a single AI system. Here, we took advantage on the one hand of the power of traditional CNN for image classification (here, VGG16). The VGG16 method processes a group of pixels and learns to represent it in different layers to understand the objects in the image around a specific location. However, the direction of the object and spatial relations between the objects are missing when applying CNN-based approaches. Therefore, in some cases, VGG16 cannot deal with the ambiguity of different viewpoints. For example, it may have an image of CRC normal gland where the lumen is around pixels [x, y], the goblet cells are around this lumen, and the nucleus are around outside creating the boundary of a gland. A CNN can easily identify the images that have similar structure features in similar locations. However, if there are any transformation or rotation (not in the same direction or degree) of these elements, for example considering the gland at different grades of CRC (dysplasia), the structure of the lumen, globet cells and nucleus are changed and each element is transformed in different ways and orientations, which may be a considerable challenge for CNN approaches. CapsNet encapsulates all information of object features detected in a vector form as output. The orientation of this capsule vector encodes the pose information of the object and its length indicates the likelihood that the object is present in the image. Due to the hierarchical architecture, CapsNet can learn the deep context of different level features making it different from VGG16 with more confidence in classification and no restriction on a specific viewpoint.

We considered ensemble learning of VGG-based and CapsNet methods here for decision-making and show an outperformance to independent learning with these methods. With the best ensemble method SVEVC, we reach an accuracy of > 93% for all classes. Our SVEVC classifier is amenable to tissues originating from different institutions that may vary based on age of tissue blocks, and other pre-analytical variables. Using the color enhancement technique proposed by Cao and colleagues^18^ differences in brightness or darkness were augmented and adjusted to allow for color differences to be incorporated into the algorithm. Beside of data augmentation for training dataset, we also applied RTS method for the augmentation of test data to reduce the uncertain cases of prediction. These augmentation techniques ensure that there is no difference in algorithm performance for the ngTMAs with different core sizes (0.6 and 1.0 mm). In addition, the RTS uses an average technique to provide an uncertainty measurement that may be informative within histopathology images. It reduces the errors of decision making when there are two classes with similar quantitative information: the number of best prediction and the average of classification score considering for all tiles of a TMA score. After applying RTS method, we obtained a group of uncertain decision where the RTS can not make the decision for the TMA core with two similar scores or the final decsion is different the prior information of the slide. All of uncertain cases will be checked by pathologist experts in the validation step.

The most common misclassification errors, were the prediction of tumor cores as other tissues and prediction of normal cores as tumor. By evaluating the incorrectly labeled tissue cores, the reasons for this misclassification are easily recognized. In the first case, tissue cores with only a few remaining cancer cells do not provide enough information for a classification of tumor. In real-life, this is not so problematic, as such tissue cores would very likely excluded from an analysis of biomarkers; such cores are considered uninformative. In 1.12% of cases, so-called “normal” cores were labeled as tumor. In most of these cases, dysplastic glands are recognized as “tumor”, despite not representing invasive cancer. In our experience, even on whole slide images, this is an extremely challenging issue, namely the differentiation between dysplastic glands found in adenomas/ precursor lesions and, especially, well-differentiated carcinomas. There have been publications investigating differences in adenoma type^38,39^, however the distinction between dysplastic lesions and cancers still remains a considerable challenge.

To summarize, the novel aspects of this study are as follows:

- We have established an automatic and effective pipeline for the classification of colorectal tissue images from high-throughput TMAs. Our method can be used after multiple sectioning of TMAs to re-assign correct labels to individual cores. It should be noted that TMA archives with well-labeled tissues and corresponding patient data may provide extensive information to help understand the biology of CRC.
- The proposed method includes a pre-processing step with tissue core extraction, color augmentation to detect and adapt the TMA core with the effect of unwanted variations due to the staining and scanning differences. Our proposed solution was applied for H&E staining image but it can be also easily extended to other immunohistochemistry stains.
- The core module of classification is based on ensemble learning methods with two differentiate neural network architectures VGG16 and CapsNet. It takes advantages of both classical VGG16 with max-pooling and hierarchical Capsnet combined with prior information about the punch location to provide a high accuracy classification algorithm. Beside of classical augmentation techniques for training dataset, we also applied the RTS for augmentation of test images in inference step to reduce the uncertain predictions.
- A large and heterogeneous real-world dataset of thousands of TMA images stained with H&E and constructed from material of 770 CRC patients diagnosed at three different institutes were used for evaluation and performance compared using different approaches. This study provides a solid reliable solution for CRC detection and a tissue screening/re-classification tool for TMA.

We will focus our future work on auto machine learning methods for the automatic selection of the different methods and hyperparameter optimization^40^, a new effective strategy for the combination of multiple models of deep learning ensemble with diverse architechtures^41^ that can be explainable classification solutions integrated with the expertise knowledge of pathologists^42^.

## Supplementary information

Supplementary Information.
