# Supplementary material for: Classification of colorectal tissue images from high throughput tissue microarrays by ensemble deep learning methods
Source: Sci Rep. 2021 Jan 27;11:2371. doi: 10.1038/s41598-021-81352-y (PMC7840737; doi:10.1038/s41598-021-81352-y)
Supplement: Supplementary file 1 — Supplementary Information. [file 41598_2021_81352_MOESM1_ESM.docx]

**Classification of colorectal tissue images from high throughput tissue microarrays by ensemble deep learning methods**

**Huu-Giao Nguyen, Annika Blank, Heather Dawson, Alessandro Lugli, Inti Zlobec**

Correspondence to:

Prof. Inti Zlobec

Institute of Pathology
University of Bern

Murtenstrasse 31

CH-3008

Email: [inti.zlobec@pathology.unibe.ch](mailto:inti.zlobec@pathology.unibe.ch)

**Keywords**: colorectal cancer, neural networks, tissue microarray, deep learning

**Supplemental Tables**

Table 1: The detail of clinicopathological data for all 3 cohorts.

|  | | Swiss Cohort  410 patients | | Canadian cohort  271 patients | | German cohort  89 patients | |
| --- | --- | --- | --- | --- | --- | --- | --- |
| **Gender** | Female | 161 | 39.27% | 62 | 22.88% | 40 | 44.94% |
|  | Male | 249 | 60.73% | 87 | 32.10% | 48 | 53.93% |
|  | No data | 0 | 0 | 122 | 45.02% | 1 | 1.12% |
|  | | | | | | | |
| **Age at diagnosis** | <50 | 32 | 7.80% | 14 | 5.17% | 12 | 13.48% |
|  | 50-64 | 108 | 26.34% | 45 | 16.61% | 39 | 43.82% |
|  | 65-74 | 116 | 28.29% | 39 | 14.39% | 19 | 21.35% |
|  | >=75 | 154 | 37.56% | 51 | 18.82% | 18 | 20.22% |
|  | No data | 0 | 0 | 122 | 45.02% | 1 | 1.12% |
|  | | | | | | | |
| **Tumor location** | Left colon | 152 | 37.07% | 74 | 27.31% | 75 | 84.27% |
|  | Right colon | 124 | 30.24% | 67 | 24.72% | 13 | 14.61% |
|  | Rectum | 108 | 26.34% | 5 | 1.85% | 0 | 0 |
|  | No data | 26 | 6.34% | 125 | 46.13% | 1 | 1.12% |
|  | | | | | | | |
| **Budding range ITBCC** ^1^ | 0-4 (Bd1) | 112 | 27.32% | 33 | 12.18% | 0 | 0 |
|  | 5-9 (Bd2) | 64 | 15.61% | 44 | 16.24% | 0 | 0 |
|  | >10 (Bd3) | 110 | 26.83% | 72 | 26.57% | 0 | 0 |
|  | No data | 124 | 30.24% | 122 | 45.02% | 89 | 100% |
|  | | | | | | | |
| **Histological Subtype** | Adenocarcinoma | 355 | 86.59% | 0 | 0 | 0 | 0 |
|  | Mucinous carcinoma | 35 | 8.54% | 0 | 0 | 0 | 0 |
|  | Others | 20 | 4.88% | 0 | 0 | 0 | 0 |
|  | No data | 0 | 0 | 271 | 100% | 89 | 100% |
|  | | | | | | | |
| **Grade** | G1-G2 | 314 | 76.59% | 149 | 54.98% | 56 | 62.92% |
|  | G3 | 88 | 21.46% | 0 | 0 | 31 | 34.83% |
|  | Others | 0 | 0 | 0 | 0 | 1 | 1.12% |
|  | No data | 8 | 1.95% | 122 | 45.02% | 1 | 1.12% |
|  | | | | | | | |
| **TNM** | I | 48 | 11.71% | 0 | 0 | 1 | 1.12% |
|  | II | 109 | 26.59% | 150 | 55.35% | 43 | 48.31% |
|  | III | 125 | 30.49% | 0 | 0 | 28 | 31.46% |
|  | IV | 121 | 29.51% | 0 | 0 | 15 | 16.85% |
|  | No data | 7 | 1.71% | 121 | 44.65% | 2 | 2.25% |
|  | | | | | | | |
| **DFS/OS 5years** | 0 (alive) | 249 | 60.73% | 127 | 46.86% | 0 | 0 |
|  | 1(dead) | 161 | 39.27% | 21 | 7.75% | 0 | 0 |
|  | No data | 0 | 0 | 123 | 45.39% | 89 | 100% |

Table 2: The detail of optical parameters for all 3 cohorts.

|  | Swiss Cohort | | German Cohort | Canadian Cohort |
| --- | --- | --- | --- | --- |
|  | Set 1 | Set 2 |  |  |
| Objective magnification | 40x | 20x | 20x | 20x |
| $\boldsymbol{\mu m/pixel}$ **X** | 0.194475 | 0.243094 | 0.243094 | 0.242792 |
| $\boldsymbol{\mu m/pixel}$ **Y** | 0.194475 | 0.243094 | 0.243094 | 0.242792 |

Table 3: The detail of number of slides and cores of each cohort using for training and two test scenarios and the information of general label (prior information of contents of the punnch).

|  | | **Swiss cohort**  **(43 slides)** | | **Canadian cohort**  **(10 slides)** | | **German cohort**  **(1 slide)** | |
| --- | --- | --- | --- | --- | --- | --- | --- |
|  |  | Number of slides | Number of cores | Number of slides | Number of cores | Number of slides | Number of cores |
| **Slide with general label** | Normal | 9 | 2737 | 2 | 295 | 1  Mixed | 165 |
|  | Other | 6 | 1443 | 3 | 448 |  |  |
|  | Tumor | 28 | 9283 | 5 | 779 |  |  |
|  | | | | | | | |
| **Training dataset** | Normal | 2 | 712 | 1 | 123 | 0 | 0 |
|  | Other | 1 | 220 | 1 | 186 |  |  |
|  | Tumor | 2 | 754 | 1 | 149 |  |  |
|  |  |  |  |  |  |  |  |
| **Test dataset of Evaluation A** | Normal | 4 | 1168 | 0 | 0 | 1 | 165 |
|  | Other | 2 | 372 |  |  |  |  |
|  | Tumor | 9 | 2612 |  |  |  |  |
|  | | | | | | | |
| **Test dataset of Evaluation B** | Normal | 3 | 857 | 1 | 172 | 0 | 0 |
|  | Other | 3 | 851 | 2 | 262 |  |  |
|  | Tumor | 17 | 5917 | 4 | 630 |  |  |

**Supplemental Figures**

**
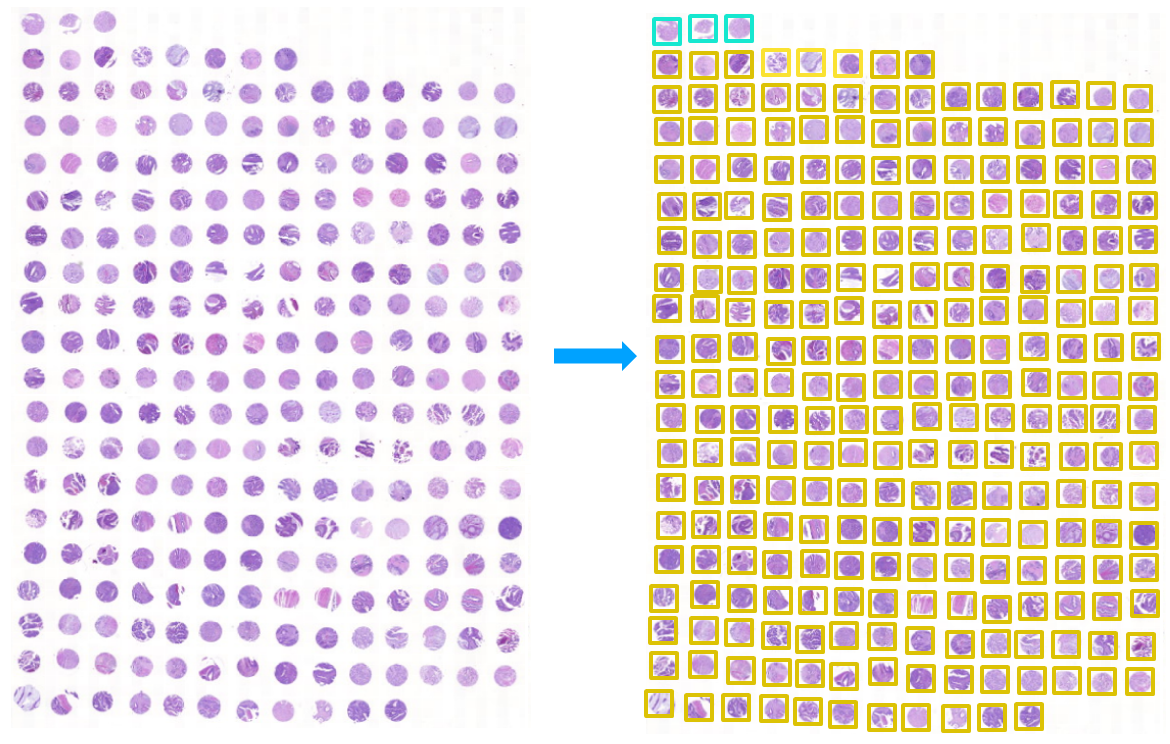
**

**Figure 1:** Example of an input ngTMA slide (left) and result of tissue core extraction (right) where: control cores in cyan, detected cores for reclassification in yellow.


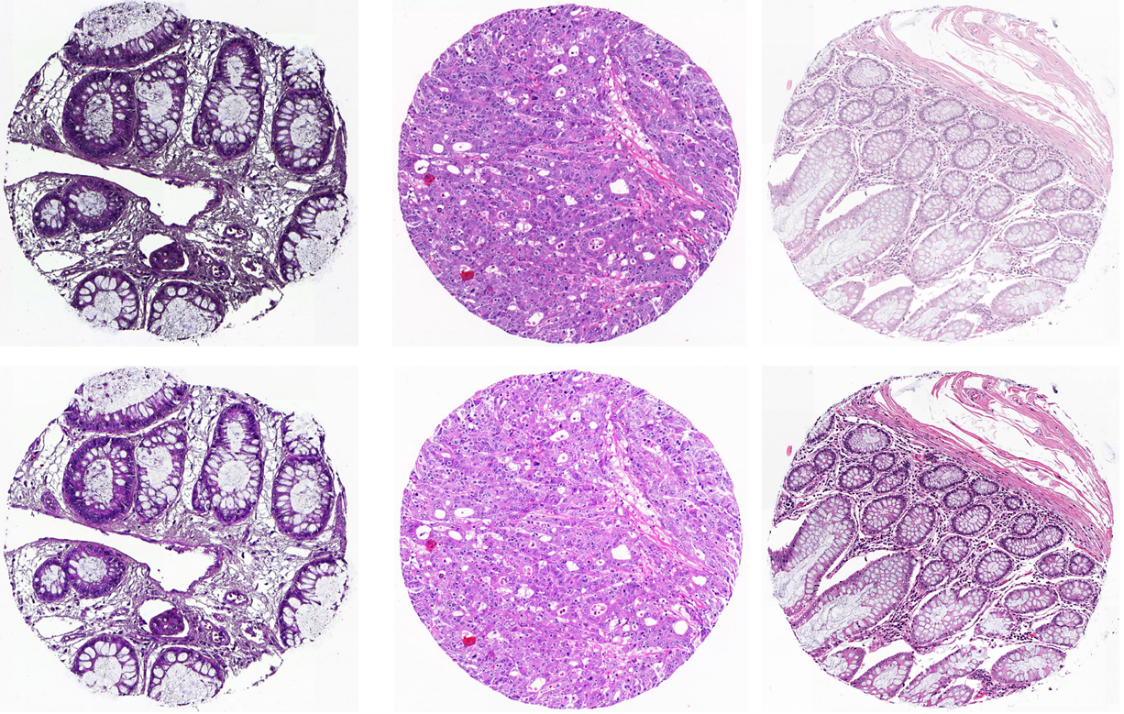


**Figure 2:** Example of different brightness levels of 3 cohorts where: Bern cohort (a), Munich cohort (b), Canadian cohort(c) in the first row and the result of contrast enhancement in the second row.


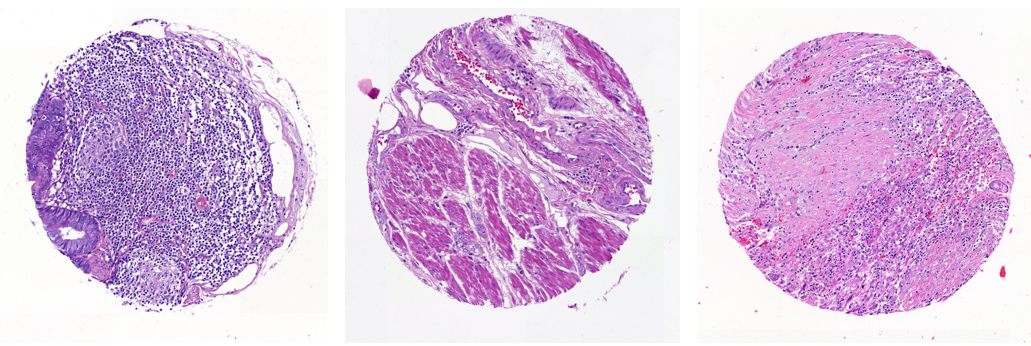


**Figure 3**: Example of uncertain cases


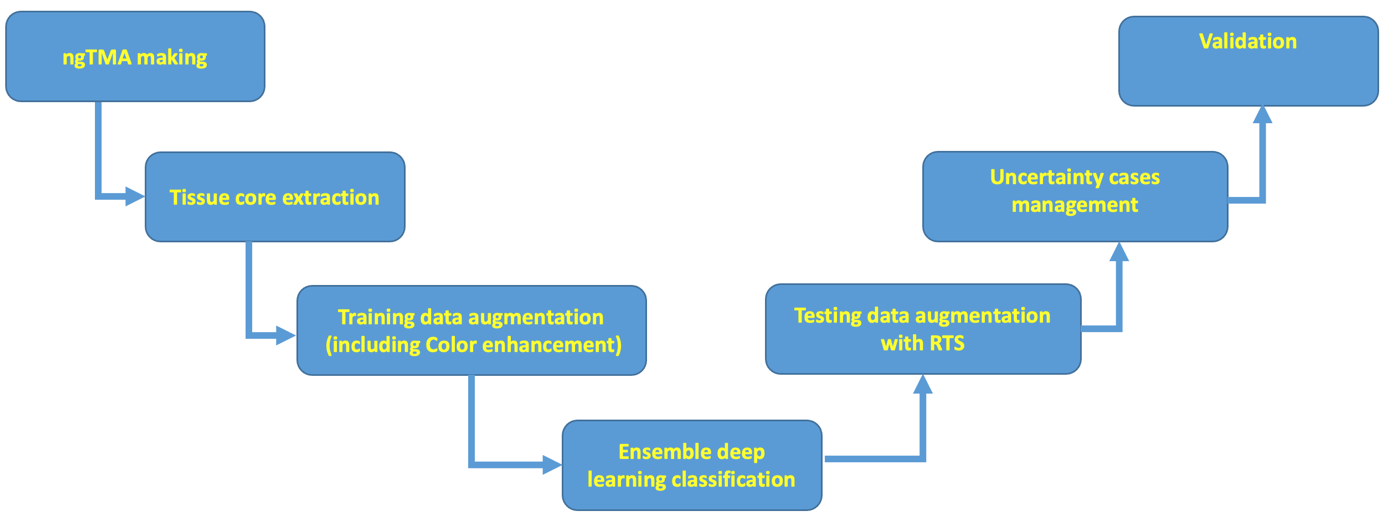


**Figure 4:** Main pipeline of the proposed method.


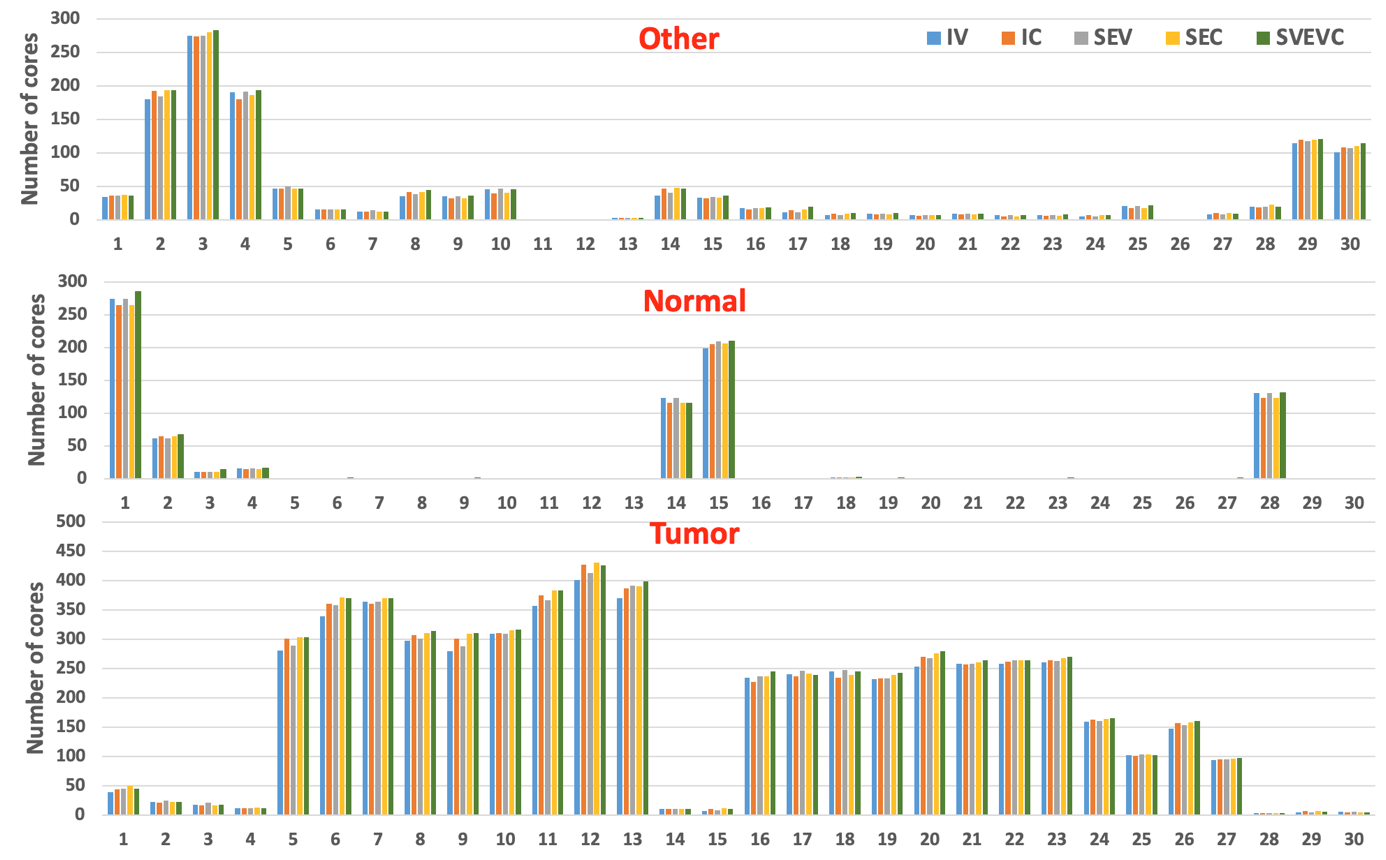


**Figure 5:** The number of correct TMA core classification for the prediction of each slide with five learning flows in evaluation B.


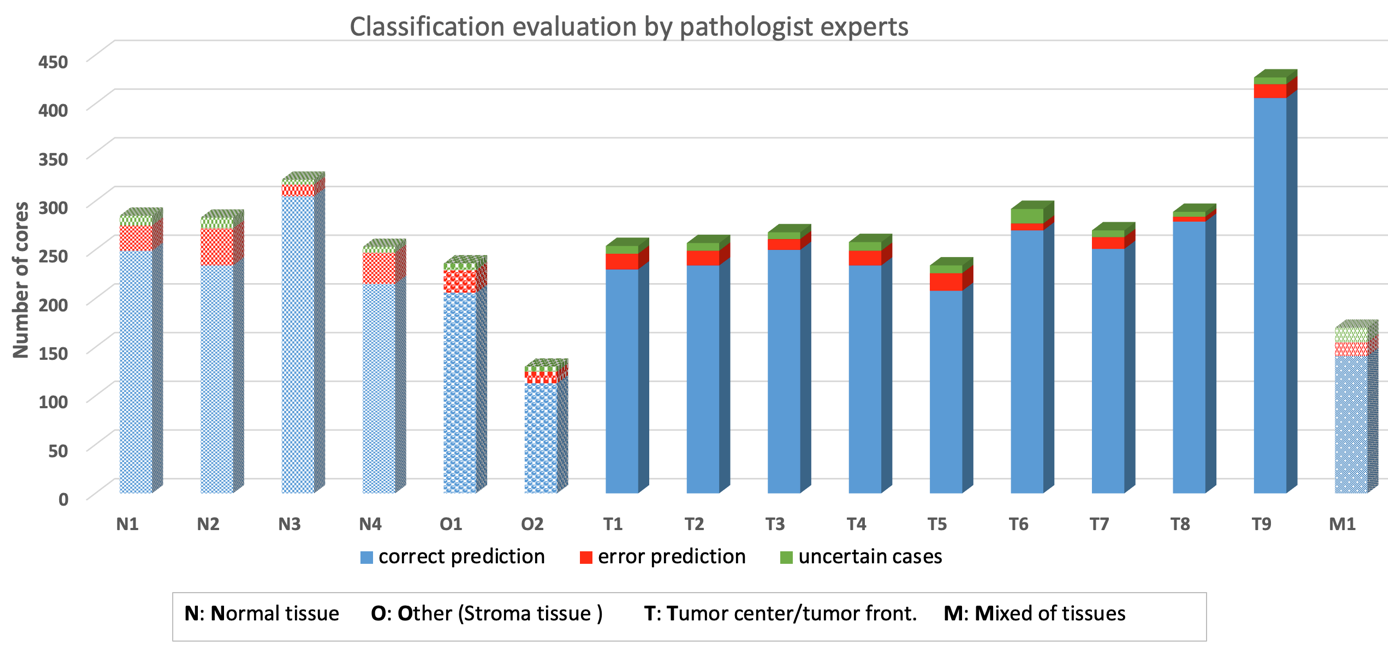


**Figure 6**: The detail of classification result per slide of SVEVC method evaluated by pathologist experts in evaluation A.

**REFERENCES**

1. Lugli, A. *et al.* Recommendations for reporting tumor budding in colorectal cancer based on the International Tumor Budding Consensus Conference (ITBCC) 2016. *Mod. Pathol.* **30**, 1299–1311 (2017).
